# Effects of Anthocyanin-rich Berries on the Risk of Metabolic Syndrome: A Systematic Review and Meta-analysis

**DOI:** 10.1900/RDS.2022.18.42

**Published:** 2022-03-31

**Authors:** Mikkel Roulund Wilken, Max Norman Tandrup Lambert, Christine Bodelund Christensen, Per Bendix Jeppesen

**Affiliations:** Department of Clinical Medicine. Aarhus University Hospital. Aarhus University. Palle Juul-Jensens. Boulevard 165. Aarhus N. Denmark.

**Keywords:** anthocyanins, berries, cardiovascular disease, lipids, metabolic syndrome, primary prevention

## Abstract

**OBJECTIVE:**

Metabolic syndrome (MetS) can lead to fatal complications, including cardiovascular disease. Emerging evidence suggests has emerged that increased fruit and vegetable intake and decreased intake of saturated fats, simple sugars, and processed foods can improve cardiovascular health. Anthocyanins (color pigments) have anti-inflammatory and antioxidant capacities but are of low bioavailability. In this systematic review and metaanalysis, we investigate the possible beneficial effects of the intake of berries high in anthocyanins on MetS risk factors. We also investigate the influences of high-density lipoprotein (HDL), low- density lipoprotein (LDL), triglycerides (TG), and total cholesterol (TC).

**METHODS:**

We identified 2,274 articles from PUBMED and EMBASE following a search input designed to include studies of interest of these, 21 met inclusion criteria.

**RESULTS:**

The studies showed an overall reduction in low-density lipoprotein (p=0.04). Increases in HDL were found with cranberry and freeze-dried berry intake during a 4-6-week intervention. No statistically significant findings were detected for fasting glucose, Hb1Ac, insulin levels, blood pressure, oxidized LDL (OX-LDL), BMI, and overall HDL.

**CONCLUSIONS:**

We conclude from this systematic review and meta-analysis that increased berry intake improves MetS key risk factors and reduces the risk of cardiovascular disease. Pronounced effects were apparent for concentrated berry products, such as freeze-dried strawberries.

## 1 Introduction

### 1.1 Cholesterol, cardiovascular disease, and metabolic syndrome

Following the current definition of metabolic syndrome (MetS) introduced in 1998, it has been estimated that more than one-fourth of the adult population worldwide is affected by MetS [1,2]. Common pharmaceuticals are currently used to treat the symptoms of MetS, including blood pressure regulators, cholesterol-lowering medications and drugs to treat type 2 diabetes mellitus (T2DM). MetS patients are recommended lifestyle changes, including increased exercise, increased intake of fruits and vegetables, and decreased consumption of simple sugars, foods high in saturated fatty acids, and processed foods [3]. Studies show that these changes cause effective improvements in their condition [4].

Several studies (including both meta-analyses and observational studies) have found correlations between increased daily intake of fruits and vegetables and improved cardiovascular health [5-7]. Likewise, some studies have investigated the effects of diets high in fruits on blood lipids and sensitivity to insulin and glucose and showed a decreased risk of cardiovascular disease (CVD) [5,6]. The mechanism behind this is complex, but it has been suggested that consumption of fruits high in anthocyanins may induce some of these beneficial effects [6,8,9].

In this systematic review and meta-analysis, we focus on studies investigating the effects of anthocyanin -rich berries on high-density lipoprotein (HDL) in patients with MetS to evaluate current evidence for beneficial effects. The possible beneficial effects on low-density lipoprotein (LDL) also has been evaluated in these studies. Through this systematic review and meta-analysis, we provide an overview of the latest evidence from randomized controlled trials (RCTs) evaluating anthocyanin-rich berries.

### 1.2 Dyslipidemia and cardiovascular disease

MetS patients are more likely to develop CVD than non-obese and non-MetS patients [10,11]. The underlying mechanisms are complex, but common to patients with CVD and MetS are decreased levels of HDL and increased levels of both LDL and TG [12,13]. MetS patients have more total body fat and also more visceral fat than normal healthy individuals [14]. This can lead to increased inflammation, in particular, a chronic state of low-grade inflammation [15]. A 2019 study showed that visceral fat, in particular, makes a greater contribution to chronic inflammation per volume than any other type of fat storage[16].

The underlying mechanism causing inflammation is not fully understood, but two mechanisms have been proposed. The first mechanism was illustrated in studies by Alvehuset al. [17] and Cao et al. [18]. The studies showed that the increase in the transcription of CC chemokine receptor 2 (CCR2), macrophage migration inhibitory factor (MIF), and tumor necrosis factor a (TNF-α), a chemokine receptor, inflammatory cytokine, and pro-inflammatory factor, was greater in visceral adipose tissue than other types of adipose storage, leading to increased inflammation [17,18]. A 2007 study by Fontana et al. demonstrated that the portal vein in severely obese individuals had a 50% higher concentration of interleukin 6 (IL-6), a pro- inflammatory factor, than the radial artery, suggesting that visceral fat has a significant effect on introducing chronic inflammation in obese and MetS patients [19].

Regardless of the mechanism, the effect of visceral fat always seems to be an increased level of circulating TNF-α and IL-6, two pro-inflammatory factors [20]. These factors may activate several inflammatory pathways to cause systematic inflammation and increased secretion of insulin which may result in lipid abnormalities, including lowered HDL and raised LDL and TG [21,22]. Therefore, it is essential to investigate how to improve blood lipids and to lower the inflammatory state of MetS patients to prevent CVD.

### 1.3 Blood lipids and cardiovascular disease

Hyperinsulinemia leads to decreased HDL and increased TG. Increased TG levels have been shown to result in more TG binding to HDL, and in turn, this decreases both HDL concentration and reverse cholesterol transport. The reason for this event is that hepatic lipases have increased affinity for TG-rich HDL particles, i.e., breaking down HDL, which results in further decreased HDL levels [23,24]. The resulting increase in TG release from HDL may cause increased risk of CVD [12].

**Figure 1.**
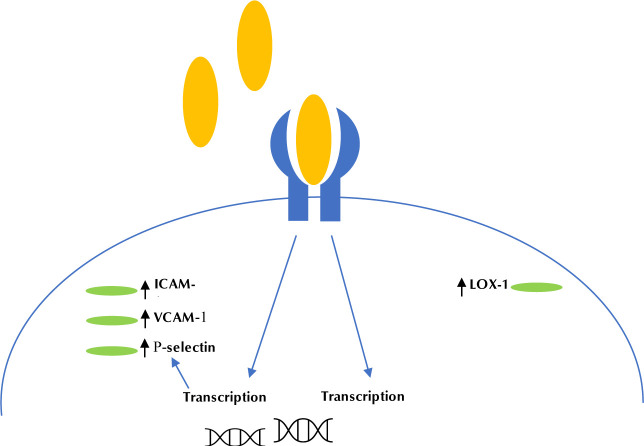
The figure illustrates the process of OX-LDL binding to LOX-1 receptor with subsequently increased transcription of LOX-1 mRNA as well as adhesion protein mRNA.

MetS patients often have elevated LDL levels and chronic low-grade inflammation, but are also likely to have elevated and unwanted production of reactive oxygen species (ROS) [15,25,26]. ROS is one of the main factors responsible for oxidizing LDL to result inoxidized LDL (OX-LDL) [27]. OX-LDL can bind to several non-native receptors on different endothelial cells including lectin-like oxidized LDL receptor 1 (LOX- 1). Binding of OX-LDL to LOX-1 induces monocyte adhesion to endothelial cells, a pre-requisite for macrophage foam cell formation in atherosclerosis, and positive feedback on the regulation of LOX-1 resulting in more LOX-1 receptors [28] (Figure 1). This process causes an upregulation of the transcription of adhesion molecules, including intercellular adhesion molecule1 (ICAM-1), vascular adhesion molecule1 (VCAM-1), and P-selectin [29].

Following upregulation of LOX-1 expression in endothelial cells, OX-LDL binds to LOX-1, which results in the initiation of endothelial apoptosis [30]. Endothelial cell apoptosis, in turn, leads to increased vascular permeability, vascular smooth muscle cell (VSMC) proliferation, and increased coagulation, thereby increasing the development of atherosclerotic lesions [31]. Atherosclerotic lesions may cause stenosis (i.e., narrowing of blood vessels), ischemia (i.e., inadequate blood and oxygen supply to an organ), and possibly ,thrombotic occlusion [32-35].

Elevated TGs affect the endothelial cells of the blood vessels in several ways. Flow-mediated dilation studies evaluating endothelial cell function have shown that elevated TG levels resulted in reduced arterial dilatation [36]. The same study concluded that chronically elevated levels of TG in hyperinsulinemic patients may lead to increased oxidative stress.

Also, postprandial hypertriglyceridemia has been shown to increase the expression of aleukocyte receptor known as lymphocyte function-associated antigen 1 (LFA-1), an essential integrin involved in the recruitment of immune cells ininflamed tissue. This mayresult in further elevated postprandial inflammation, imposing additional risk of CVD on the patient [37].

Furthermore, TG intake has been shown to affect hormonal regulation, resulting in elevated resistin levels, for example [38]. Resistin has been shown to increase transcriptional levels of pro-inflammatory cytokines, including TNF-α, interleukin1 (IL-1), IL-6, and IL-12. We know TNF-α and IL-6 can decrease HDL levels [39], and IL-12 is a proinflammatory cytokine that further elevates the inflammatory state.

In summary, dyslipidemia has devastating effects on health by increasing CVD risk, as discussed above. Therefore, it seems essential to improve the lipid profile of dyslipidemic MetS patients, a patient group that is constantly increasing in number. Global interest in ameliorating and possibly preventing CVD non- pharmaceutically has been sparked, and anthocyanins have received a great deal of interest as a potential CVD treatment.

### 1.4 Anthocyanins and their effects on high-density lipoproteins

A 2014 study by Yanna Zhu et al. showed how 320 mg/d of anthocyanins derived from bilberries and black currants resulted in a significant increase in HDL in hypercholesterolemic subjects following a 24- week intervention period [9]. The mechanism behind the improved HDL concentrations has not been fully elucidated. The authors suggest that anthocyanins or their derivatives may lead to the activation of serum paraoxonase and arylesterase 1 (PON1), which prevents HDL from being oxidized and broken down, thus increasing HDL’s cholesterol efflux capacity allowing an efficient reverse cholesterol transport [9].

Also, a 2015 in vivo study using mice by Nicholas Farrell et al. showed how mice fed an anthocyanin-rich diet had no significant serum HDL changes, but showed gene expression alterations that were associated with improved liver and HDL functions [40]. Hepatic and intestinal mRNA changes in mice fed anthocyanin-rich diets had increased transcription of ApoA1, PON1, serum amyloid A1, lecithin cholesterol acyltransferase, and apolipoprotein J, all of which led to improved HDL function. Also, a reduction in serum chemokines and ligand 2 (CCL2) was seen, which represented a lowered state of inflammation.

In a 2013 study by Kianbakht et al. much lower anthocyanin doses were used resulting in significantly increased HDL serum concentrations [41]. In this study, a total of only 9.8 mg/d of whortleberry-extracted anthocyanins were given daily for two months. The intervention group showed a 37.5% increase in serum HDL in the post-intervention period relative to baseline values.

### 1.5 Anthocyanin and their effects on low-density lipoproteins

An RCT from 2014 conducted by Rasool Soltani et al. showed a significant decrease in LDL levels in hypercholesterolemic subjects [42]. The intervention group (n=25) consumed a 45 mg whortleberry anthocyanin capsule twice daily for 4 weeks. The LDL serum levels were lowered by a mean of 11.44±3.28 mg/ dl.

Likewise, a 2008 study by Lee et al. on persons with T2DM with elevated LDL (mean LDL of 127.61±29.93 mg/dl) showed a significant LDL decrease following a 12-week intervention period [43]. The intervention group received 500 mg cranberry extract 3 times daily for a period of 12 weeks. After intervention, their LDL levels had decreased to a mean concentration of 112.14±29.93 mg/dl. The exact anthocyanin dose was not determined, but cranberries are regarded as being among the berries with the highest anthocyanin content [44].

To date, no definitive mechanism has been determined as to how anthocyanin consumption can result in lowered LDL levels. However, a 2009 study suggests that anthocyanins can impede cholesteryl ester transfer protein (CETP), a deactivation that is associated with decreased formation of both LDL, very low-density lipoproteins (VLDL), and intermediate- density lipoproteins (IDL) [8].

A 2018 cellular study by Kimet al. showed that Caco- 2 cells treated with anthocyanin-rich blackcurrant extract showed an upregulation of the low-density lipoprotein receptor, ultimately lowering LDL. It is believed that a similar effect occurs in humans [45].

### 1.6 Anthocyanins and their effects on triglycerides

In 2015, Pei-Wen Zhang et al. showed that TG serum levels significantly lowered by as muchas 17.27% after 12 weeks of blueberry consumption. The underlying mechanism is not fully understood, but a potential mechanism was proposed by Dan Li et al. in 2015. They suggested that anthocyanins may result in a reduction in serum apolipoprotein-B (ApoA-B) and apolipoprotein-C (ApoA-C) containing TG-rich particles [46].

Also, Honghui Huoet al. showed that levels of adipose triglyceride lipase (ATGL) decreased in 3T3-L1 adipocytes aftercyanidin-3-O-β-glucoside (C3G) treatment [47]. The 3T3-L1 adipocytes were treated with C3G resulting in attenuated high-glucose- promoted O-glycosylation of transcription factor FoxO1, which in turn, resulted in decreased expression of ATGL, suggesting a triglyceride-lowering mechanism. Table 1 summarizes information on possible mechanisms to improve blood lipid profile.

**Table 1.** Summary of possible mechanisms underlying blood lipid improvements seen in preclinical and clinical studies

Authors	Treatment/ dose	Type of study	Number of participants	Potential mechanism	Health benefits
Yanna Zhu et al. (2014) [9]	320 mg/d anthocyanins from bilberries and black currants	Human study, double-blinded, RCT	122 hypercholesterolemic patients	Activation of PON1 HDL oxidization and breakdown ReCT	Increase in serum HDL and possible decrease in serum LDL
Nicholas Farrell et al. (2015) [40]	Anthocyanin-rich diet	Mouse study	36 male Apo-/-mice (24 on anthocyanin diet, 12 on control diet)	ApoA1 transcription PON1 transcription Serum amyloid A1, lectin cholesterol acyltransferase transcription Apolipoprotein J transcription Chemokines and serum CCL2	Improved HDL function and lowered inflammation
Qin, Y. et al. (2009) [8]	320 mg/d anthocyanins	Human study, double blinded, RCT	20 dyslipidemic subjects	CETP activity	Decreased formation of LDL, VLDL, and IDL
Erl-Shyh Kao et al. (2009) [48]	0.05-0.2 mg/ml anthocyanins from Hibiscus	J774A.1-cells treated with OX-LDL to induce foam cell formation		CD36 Foam cell formation	Reduction in foam cell formation
Kim et al. (2018) [45]	50 or 100 gg/ml anthocyanins from black currants	Caco-2 cells		Low-density lipoprotein receptor	Increased cellular uptake of LDL resulting in decreased serum LDL levels
Pei-Wen Zhang et al. (2015) [49]	320 mg/d blueberry and black currant anthocyanin extract	Human study, double blinded, RCT	74 (39 men and 35 women)	ApoA-B ApoA-C	Reduction in CVD and CHD risk[50,51]
HonghuiHuo et al. (2012) [47]	Cyanidin-3- glucoside treatment	3T3-L1 adipocytes		ATGL activity	Decreased serum TG levels

**Legend**: ApoA1 - apolipoprotein A1, ATGL - adipose triglyceride lipase, CETP - cholesteryl ester transfer protein, CCL2 - chemokines and ligand 2, CVD - cardiovascular disease, HDL - high-density lipoprotein, IDL -intermediate-density lipoproteins, LDL - low-density lipoproteins, PON1 -paraoxonase and arylesterase 1, ReCT- reverse cholesterol transport, RCT - randomized controlled trial.

### 1.7 Anthocyanins and their effects on insulin secretion and sensitivity

In 2010, April J. Stull et al. carried out a study to investigate the effect of daily blueberry consumption on whole-body insulin sensitivity [52]. In this study, 32 obese, non-diabetic, insulin-resistant subjects were recruited, and a significant increase in insulin sensitivity was seen in the intervention group. No mechanism for the increase in insulin sensitivity has yet been determined in human subjects, but a mechanism has been proposed in mice, where bilberry anthocyanins were reported to have activated adenosine monophosphate-activated protein kinase (AMPK) in mouse skeletal muscles, liver, and white adipose tissue [53]. This activation apparently caused an upregulation of glucose transporter four in skeletal muscles and white adipose tissue, while down regulating glucose production in the liver.

### 1.8 Anthocyanins and their antioxidant capacities

Only a few studies evaluate berry antioxidants in humans, but these show promising results. A 2015 clinical trial by Sergio Davinelli et al. showed that 486 mg of anthocyanins daily significantly reduced levels of OX-LDL post intervention [54]. However, following a literature search, it is evident that there are no studies demonstrating that the anthocyanins administered prior to their metabolism in humans are responsible for the postulated antioxidant function of anthocyanins . Therefore, it might be their metabolites that exert antioxidative effects. In this regard, it is notable that polyphenols are among the major metabolites. Regardless of whether the parent anthocyanins themselves or their metabolites are responsible for exerting effects, several in vitro, in vivo, and clinical studies have demonstrated positive effects fromanthocyanin consumption [55].

## 2 Methods

### 2.1 Search strategy and study selection

This review includes articles identified by systematic search as well as other articles identified by the research team outside the search strategy. These articles include studies carried out in vitro prior to human studies to identify possible benefits of anthocyanins. The meta-analysis includes only articles identified by the systematic search strategy. In this meta-analysis, both PUBMED and EMBASE have been searched systematically for randomized clinical trials up to April 31, 2020. Appendix 1 shows

the terms used for PUBMED and EMBASE searches. The study was registered on Prospero with ID CRD42020181167.

Human participants with diagnosed MetS (MetS) or fulfilling 3 out of 5 diagnostic criteria for MetS diagnosis were included in the meta-analysis [1]. The criteria for MetS diagnosis were applied according to the International Diabetes Federation Guidelines, updated in 2020 [56]:

- Waist circumference >80 cm
- TG level >150 mg/dl
- HDL <40/50 mg/dl
- Blood pressure >130/85
- Fasting glucose >100 mg/dl

Despite BMI not being required for MetS diagnosis, BMI above 25 has shown similar systemic consequences as MetS including the development of T2DM [57]. Likewise, if the articles did not meet the criteria, we estimated that if participants had BMI >27, their waist circumference would be above 80 cm.

The following inclusion criteria also had to be met:

- Human studies
- Adults >18 years of age
- Intervention length >4 weeks
- Reported mean end values at the end of the intervention with their corresponding SD, SEM or confidence intervals (CI)
- The intervention products were provided by the study personnel to the participants
- Publications dating from 1970 or more recently
- Intervention products included fresh, freeze- dried, juices and extracts from berries with proven anthocyanin content.

The exclusion criteria were as follows:

- No MetS patients
- No control group
- The control group had diseases different from the intervention group
- Alcohol interventions (red wine, etc.)
- BMI <25
- Intervention length < 4 weeks
- Interventions consisting of recommendations, telephone calls, etc.
- Reviews
- Observational studies
- Meta-analyses
- Data missing or not presented

All articles were evaluated based on title, abstract, and full text by two independent reviewers. The outcome of interest in this systematic review and metaanalysis was HDL level. However, an evaluation of LDL and TC as well as OX-LDL, BMI, blood pressure, fasting glucose, Hb1Ac, and insulin levels also had been carried out based on the included studies. If the publication appeared prior to 1970, the study was not included.

### 2.2 Quality assessment

Two authors (Wilkins and Christiansen) independently assessed all articles following the PUBMED and EMBASE search. All included articles went through a risk of bias assessment by means of the Cochrane collaboration’s tool that includes the following criteria:

- Random sequence generation
- Allocation of concealment
- Blinding of participants and personnel
- Blinding of outcome assessment
- Incomplete outcome data
- Other possible causes for bias

In the case of divergence between the assessments by the two authors, a third author (Lambert) independently assessed the articles to reach consensus. The quality of included studies was evaluated using the risk of bias assessment tool from the Cochrane Handbook for Systematic Reviews of Interventions (version 5.3.0). It includes tools for selection bias (random sequence generation and allocation concealment), performance bias (blinding of participants and personnel), detection bias (blinding of outcome data), attribution bias (incomplete outcome data), reporting bias (selective reporting), and other sources of bias.

### 2.3 Data extraction

Only studies publishing mean ±SD, mean ±SEM, or a 95% CI for endpoint values were used. We used Cochrane Handbook version 5.1.0 section 7.7.3.2 to convert mean ±SEM and CI to mean ±SD. All HDL, LDL, TC, and other values were converted to mg/dl. Fasting glucose, Hb1Ac, insulin levels, and BMI were converted to mg/dl, percent, mU/l, and kg/m2, respectively. Also, subgroup analyses were performed for BMI >30, HDL levels, anthocyanin doses, intervention length, and kind of berry used for intervention.

### 2.4 Statistical analysis

Mean ±SD values for end values within both the intervention and the control group were all pooled in Review Manager (RevMan) 5.3 (the Cochrane Collaboration’s software) to analyze and evaluate the effects on HDL, as well as other parameters, in both the control and intervention group following the intervention period. RevMan includes tools for protocol and review preparation, assessing characteristics of studies, and comparison tables. It has tools to perform meta-analyses and can present results graphically.

With regard to one study (P. J. Curtis et al. 2019), the 95% CI for HDL concentrations post intervention with one cup of blueberries measured in mmol/l was not presented with adequate decimals to calculate a ±SD of more than 0 following a conversion from mmol/l to mg/dl [58]. The 95% CI presented was (1.2, 1.2). To ensure a ±SD for the analysis, the CI used was set to (1.21, 1.22) (Appendix A2).

To evaluate and determine heterogeneity, we used the measure of inconsistency, where I2>50% was considered as substantial heterogeneous. We used the random effect model to determine statistical significance. Weighted mean difference and 95% CI were obtained from the pooled studies and visualized in forest plots to illustrate the obtained data. With regards to publication bias, the shape of the funnel plot indicated a certain asymmetry between the differences in HDL levels when anthocyanin-rich berry administration was compared with placebo. This appears more pronounced in smaller studies and becomes less prominent with larger scale trials which seemed to be more robust. It indicates the presence of systematic differences between smaller and larger studies, with a higher possibility of bias in smaller studies. However, publication bias was not confirmed by Egger's test (p=0.47) (Appendix A3).

## 3 Results

### 3.1 Selection of studies

**Figure 2.**
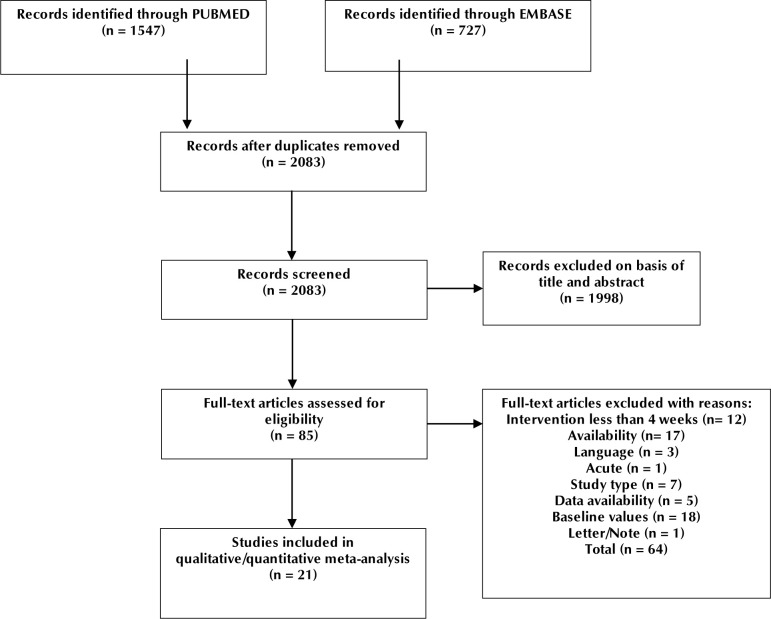
Study flow diagram showing the different steps in screening of articles obtained from PUBMED and EMBASE and the reasons for exclusions

We identified 2,274 articles by searching PUBMED and EMBASE, 21 of which have been included in this meta-analysis. After removing duplicates, screening of abstracts, and screening of full texts, 2,253 articles were discarded leaving a total of 21 articles to be included in the study (Figure 2). Six 6 studies did not include endpoint data for evaluated parameters, but authors were contacted; one author responded and provided the data, the remaining 5 have been excluded.

All 21 studies were randomized controlled trials or randomized crossover studies. A total of 25 interventions provided a total of 1,355 participants in this meta-analysis. Studies included participants with dyslipidemia [41,59,68,69,60-67], MetS [58,70,71], T2DM [46,72-75], and nonalcoholic fatty liver disease (NAFLD) [49]. All participants in this analysis who had not already been diagnosed with MetS were evaluated for MetS; they were regarded as MetS patients if they fulfilled three out of five criteria

Different types of intervention and placebo were administered in the studies. The intervention duration varied from four weeks to six months. Prior to intervention, we observed no statistically significant differences between the placebo group and intervention group in all studies.

**Figure 3.**
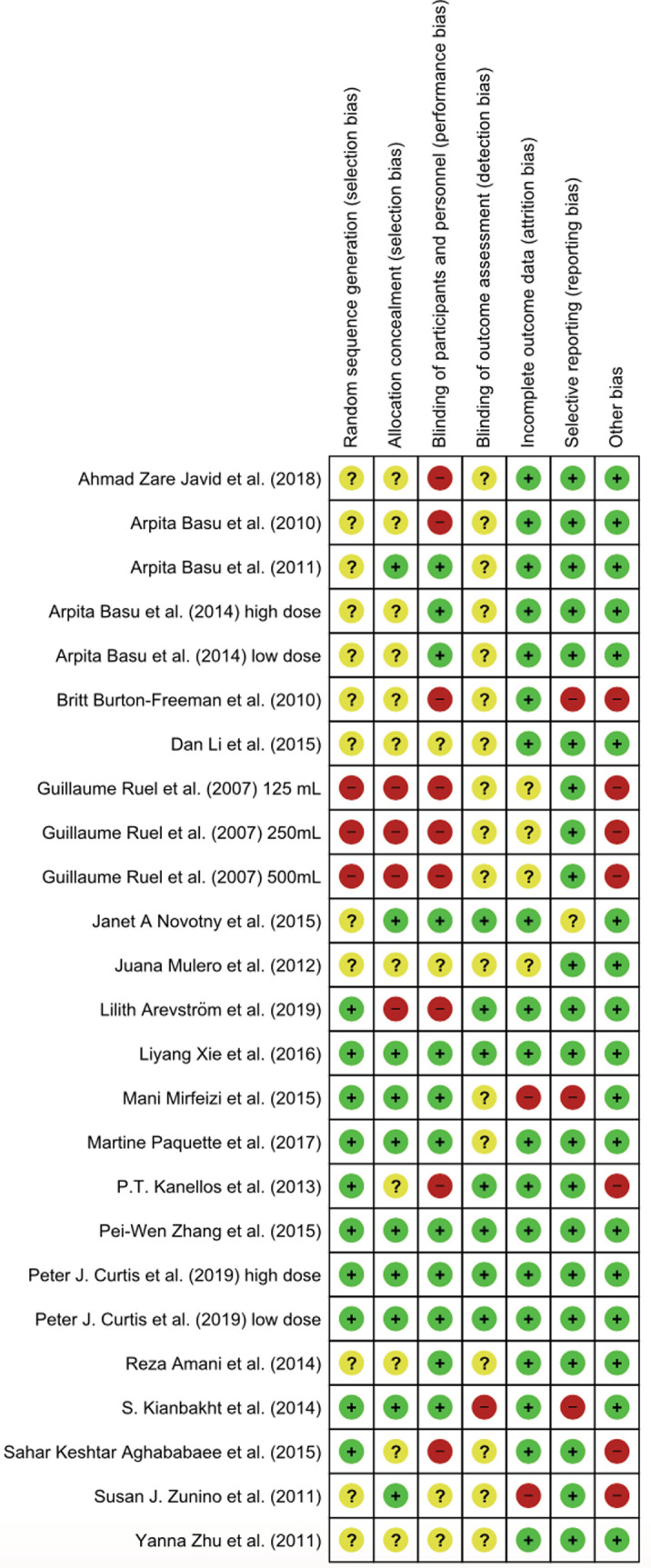
Risk of bias summary of authors. Green = low risk of bias, red = high risk of bias.

All studies included in the meta-analysis were evaluated for risk of bias (Figure 3). The differences between the studies were as follows:

- Ten studies had no information on the method of randomization [46,58,61,62,66,68-71,75].
- Seven studies had no information on allocation concealment [46,49,68,70,71,73,75].
- Four studies were open-labelled [59,67,70,73].
- Twelve studies had missing information on blinding or blinding method[41,46,74,75,59,63, 65,66,68-70,72].
- Seven studies had missing or no information on blinding of personnel[58-60,63,64,67,68].

There were also other causes for possible bias in the following studies:

- One study had 45 participants in the control group, but only showed data on three[74].
- One study did not provide the amount of anthocyanin in their product; so this was estimated [66].In one study, there was a significant difference in HDL levels between the intervention and placebo group in the preintervention period [60] (Table 2.

**Table 2.** Summary of studies included in the meta-analysis

Study	Study type	Participant characteristics	Number of participants (age)	Duration	Intervention	Placebo	Country
Aghababaee (2015) <span style=”baseline”> 59]</span>	RCT	Dyslipidemia	Inv: 36 (45.08±7.58) Con: 36 (45.61±8.69)	8 weeks	300 ml/d blackberry juice with pulp	Usual diets	Iran
Arvestöm (2018) <span style=”baseline”>[67]</ span>	RCT	Dyslipidemia	Inv: 25 (66 (62-71)) Con: 25 (68 (62-74))	8 weeks	10 g freeze-dried bilberries in capsules	Capsules with bilberry flavor	Sweden
Amani (2014) <span style=”baseline”>[72]</ span>	RCT	T2DM	Inv: 19 (51.9±8.2) Con: 17 (51.1±13.8)	6 weeks	Freeze-dried strawberry beverages (25g/d powder)	Isocaloric drink with strawberry flavor	Iran
Burton-Freeman (2010)<span style=”baseline”>[60]</ span>	RC	Dyslipidemia	Inv: 12 Con: 12 (50.9±15.0)	2x6 weeks	Strawberry drink containing 10 g/d freeze- dried strawberry	Drink matched in energy and macronutrients	USA
Basu (2010) <span style=”baseline”>[70]</ span>	RCT	MetS	Inv: 15 (45.0±3.0) Con: 12 (48.0±5.3)	8 weeks	Strawberry drink containing 50 g/d freeze- dried strawberry	4 cups of water	USA
Basu (2011) <span style=”baseline”>[71]</ span>	RCT	MetS	Inv: 15 Con: 16 (52.0±8.0)	8 weeks	480 ml/d cranberry juice	480 ml/d placebo drink	USA
Basu (2014) <span style=”baseline”>[61]</ span>	RCT	Dyslipidemia	LD-FDS: 15 (50±10) LD-C: 15 (48±10) HD-FDS: 15(49±11) HD-C: 15 (48±10)	12 weeks	LD-FDS: 25 g/d HD-FDS: 50 g/d Freeze-dried strawberry powder	Red food color, strawberry- flavored, fibers from vegetables and gums	USA
Curtis (2019) <span style=”baseline”>[58]</ span>	RCT	MetS	Inv 1: 39 (62.6±7.2) Inv 2: 37 (63.0±5.9 Con: 39 (62.9±8.1)	6 months	Inv 1: % cup blueberries Inv 2: 1 cup blueberries	Isocaloric and macronutrientmatching food	UK
Kanellos (2014) <span style=”baseline”>[73]</ span>	RCT	T2DM	Inv: 26 (63.7±6.3) Con: 22 (63±8.5)	24 weeks	36g/d of Corinthian raisins,fewer fruits and vegetables than usual	Usual diet,fewer grapes and raisins	Greece
Kianbakht (2014) <span style=”baseline”>[41]</ span>	RCT	Dyslipidemia	Inv: 40 (51.3±15.27) Con: 40 (55.8±13.28)	2 months	4 times daily consumption of capsules containing 2.45 mg anthocyanin each	4 times daily consumption of placebo capsule containing no anthocyanin	Iran
Li (2015)<span style=”baseline”>[46]</ span>	RCT	T2DM	Inv: 29 (57.6±3.4) Con: 29 (58.1±2.3)	24 weeks	160 mg anthocyanin capsule twice daily	Placebo capsule containing no anthocyanin twice daily	China
Mirfeizi (2016)<span style=”baseline”>[74]</ span>	RCT	T2DM	Inv: 30 (55±10) Con: 45 (54±12)	90 days	1 g/d of whortleberry	Starch capsules	Iran
Mulero (2012)<span style=”baseline”>[68]</ span>	RCT	Dyslipidemia	Inv: 18 Con: 15	6 months	300 ml citrus juice including aronia berry extract once daily	300 ml citrus juice once daily	Spain
Novotny (2015)<span style=”baseline”>[62]</ span>	RCT	Dyslipidemia	Inv: 29 (49.8±11.3) Con: 27 (51.3±11.1)	8 weeks	240 ml/d low-calorie cranberry juice	Placebo product, unclear masking	USA
Paquette (2017) <span style=”baseline”>[63]</ span>	RCT	Dyslipidemia	Inv: 20 (57±1) Con: 21 (60±1)	6 weeks	Beverage containing 333 mg strawberry and cranberry polyphenols	Flavor- matched drink containing no polyphenols	Canada
Ruel (2008) <span style=”baseline”>[64]</ span>	RC	Dyslipidemia	Inv 1: 30 Inv 2: 30 Inv 3: 30 Con: 30 (51±10)	4x4 weeks	Inv 1: 125 mlcranberry juice + 375ml placebo drink Inv 2: 250 mlcranberry juice + 250 ml placebo drink Inv 3: 500 mlcranberry juice	Control: 500 ml placebo drink	Canada
Xie (2017) <span style=”baseline”>[65]</ span>	RCT	Dyslipidemia	Inv: 25 (32.6±2.6) Con: 24 (37.4±3.0)	12 weeks	2 capsules daily providing 500 mg/d of aronia extract	2 placebo capsules identical in color and appearance daily	USA
Javid (2018) <span style=”baseline”>[75]</ span>	RCT	T2DM	Inv: 9 (57.88±6.03) Con: 12 (53.60±6.23)	8 weeks	200 ml/d cranberry juice	No placebo product	Iran
Zhang (2015) <span style=”baseline”>[49]</ span>	RCT	NAFLD	Inv: 37 (44.9±7.5) Con: 37(46.9±7.7)	12 weeks	320 mg anthocyanin- containing capsules from bilberry and black currant	Placebo capsules	China
Zhu (2011) <span style=”baseline”>[69]</ span>	RCT	Dyslipidemia	Inv: 73 Con: 73	12 weeks	320 mg anthocyanin/day	Placebo capsule containing no anthocyanin	China
Zunino (2011) <span style=”baseline”>[66]</ span>	RC	Dyslipidemia	Inv: 20 Con: 20 Male: 29.4+6.6 Female: 31.8±11.4	7 weeks	Diets provided 7/d/week containing 4 servings strawberries/day	Diets provided 7/d/week containing no strawberry powder	USA

**Legend**: Age given with ±SD, SEM, or range if provided. If age was not provided in articles per group for intervention and control groups, age shown is for both groups and sexes combined. Abbreviations: Inv -intervention group, Con -control group, LD-FD -low-dose freeze-dried strawberries, HD-FDS -high-dose freeze-dried strawberries, LD-C -low-dose freeze-dried strawberry control group, HD-C -high-dose freeze-dried strawberry control group, RC - randomized crossover, RCT - randomized controlled trial, T2DM - type 2 diabetes mellitus.

### 3.2 Effects of berries high in anthocyanins on HDL

**Figure 4.**
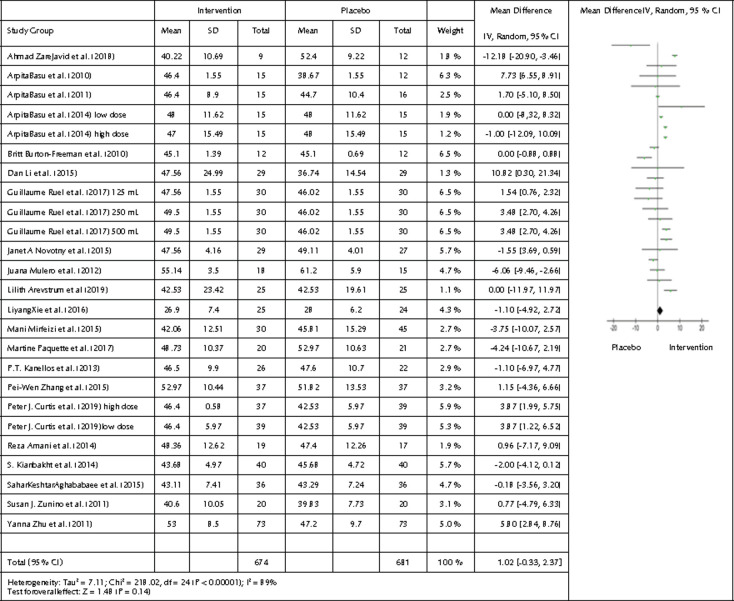
Forest plot showing the effects of the intervention included in the meta-analysis on HDL levels. Data are presented as mean difference and 95% CI using a random-effect model.

A comparison between all 1355 participants from the 21 studies and 25 interventions was made. HDL endpoint values from the intervention group were compared with the HDL endpoint values from the control group. To illustrate the comparison, we created a forest plot (Figure 4). There was no statistical significance, but we observed a tendency towards a beneficial effect of HDL concentrations following the intervention period (p=0.14, 95% CI -0.33, 2.37, and I2 of 89% (p<0.00001) showing significant heterogeneity).

**Figure 5.**
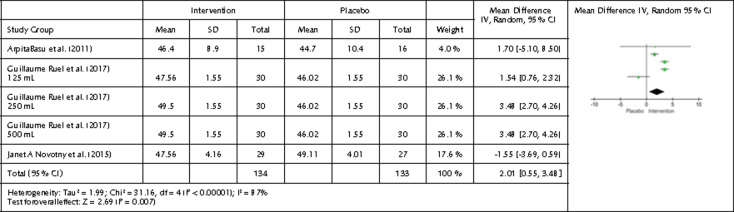
Forest plot showing the effects of cranberry intervention on HDL levels. Data are presented as mean difference and 95% CI using a random-effect model.

We performed a subgroup analysis on cranberry interventions and the effects on HDL. We obtained a statistically significant result from three studies, five interventions, and 267 participants. After intervention, HDL levels for the intervention group were significantly higher, namely 2.01 mg/dl higher than in the placebo group (p=0.007, 95% CI 0.55, 3.48, and I2 of 87%) suggesting that cranberries can increase HDL in MetS patients (Figure 5). Relative to other berries high in anthocyanins, cranberries may contain more readily bioavailable anthocyanins than other berries, which may have increased HDL in studies using cranberries to a greater degree than in those using other fruits [76].

**Figure 6.**
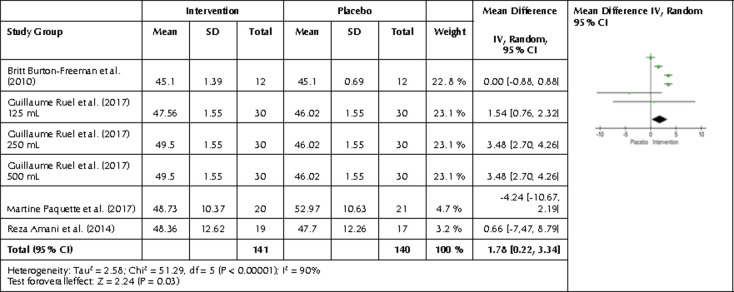
Forest plot showing the effects of intervention length of 4-6 weeks on HDL. Data are presented as mean difference and 95% CI using a random-effect model.

Following a categorization of intervention length, four studies with a total of six interventions and 281 participants showed an increase in HDL levels for the intervention group following intervention duration of 4-6 weeks. A slight increase of 1.78 mg/dl was observed with a p-value of 0.03 and 95% CI0.22, 3.34 with I2 = 90% (Figure 6). No effect was seen after an intervention shorter than 4-6 weeks. No studies lasting for more than six weeks showed any increase, but this may be due to poor adherence of participants, i.e., participants becoming less adherent to consuming the provided anthocyanin products after an extended time period.

### 3.3 Effects of berries high in anthocyanins on LDL

**Figure 7.**
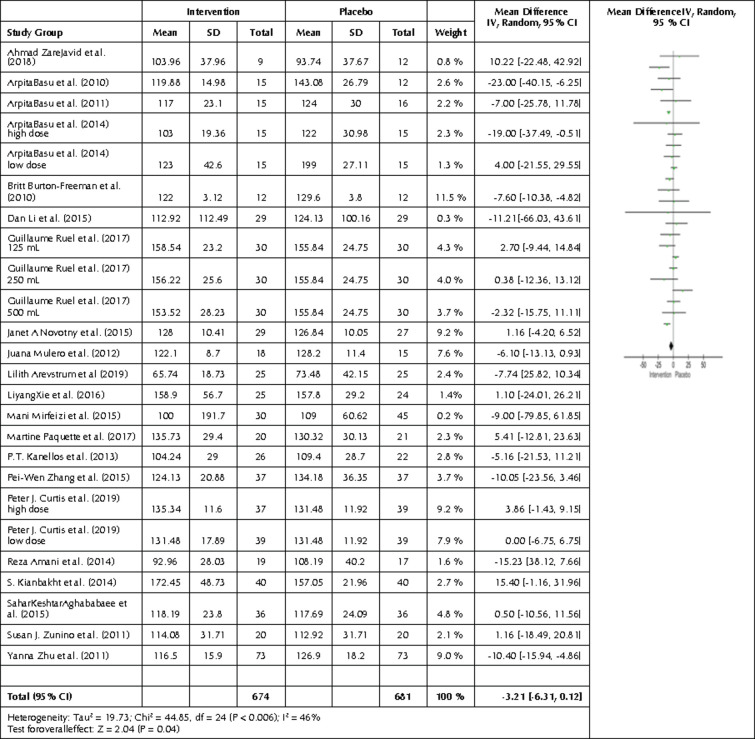
Forest plot showing the effects of the intervention included in the meta-analysis on LDL levels. Data are presented as mean difference and 95% CI using a random-effect model

We also carried out a comparison between the intervention and control group regarding LDL end values. All 21 studies and 25 interventions were compared by creating a forest plot (Figure 7), showing significantly lower LDL levels in the intervention compared to the control group (p=0.04). The mean difference was -3.21 mg/dl with a 95% CI -6.31, -0.12, and I2 of 46% (p=0.006), showing significant heterogeneity.

**Figure 8.**
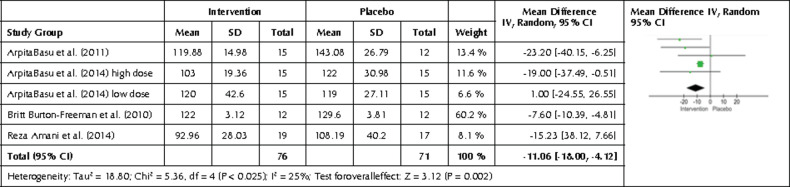
Forest plot showing the effects of freeze-dried strawberry interventions on LDL levels. Data are presented as mean difference and 95% CI using a random-effect model.

We performed a subgroup analysis on freeze- dried strawberry interventions and their effects on LDL levels. We found a statistically significant mean difference of -11.06 mg/dl in the intervention group after intervention (p= 0.002, 95% CI-18.00, -4.12) (Figure 8).

### 3.4 Effects of berries high in anthocyanins on TC

**Figure 9.**
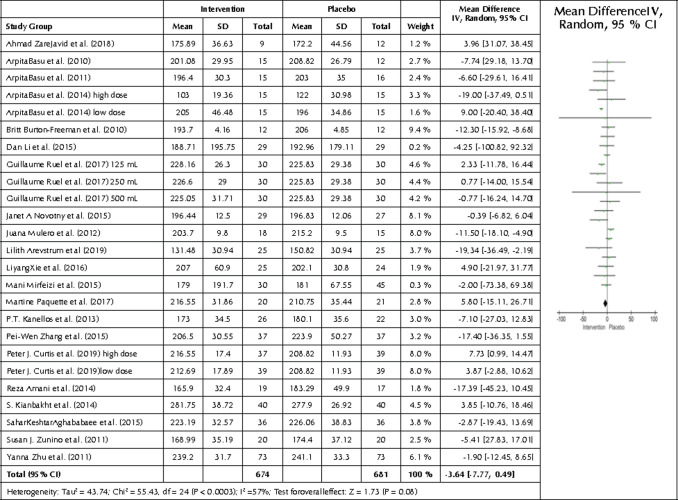
Forest plot showing the effects of the intervention included in the meta-analysis on TC levels. Data are presented as mean difference and 95% CI using a random-effect model.

A forest plot was also created from the TC end values from the 21 studies and 25 interventions (Figure 9). The forest plot showed no statistically significant differences between end values for the intervention and the control group (p=0.08). However, a tendency towards lower TC values was seen in the intervention group showing a mean difference of -3.64 mg/dl with a 95% CI -7.77, 0.49, I2 of 57%, and p=0.0003, showing significant heterogeneity.

**Figure 10.**
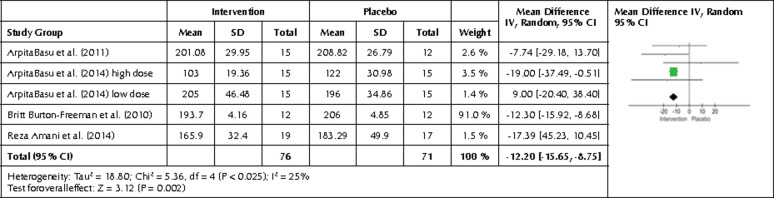
Forest plot showing the effects of freeze-dried strawberry interventions on TC levels. Data are presented as mean difference and 95% CI using a random-effect model.

Four studies and five interventions investigated freeze-dried strawberries and showed a decreasing effect on TC in the intervention group with a similar p-value of <0.00001. The mean difference between the placebo and intervention group was -12.20 mg/dl (95% CI-15.65, -8.75, and I2 of 0%) (Figure 10). Table 3 contains the results of the subgroup analyses.

**Table 3.** Subgroup analysis of pooled data from the 21 studies included

Subgroup parameter	Pooled intervention group(n)	Pooled control group(n)	Mean difference	P-value	95% CI	Heterogeneity (I2)	p-value for heterogeneity
Fasting glucose levels	335	345	3.29 mg/dl	0.09	-0.53, 7.11	92	<0.00001
Hb1Ac	225	241	0.04%	0.45	-0.06, 0.13	37	0.12
BMI change	233	251	-0.69 kg/m2	0.02	-1.27, -0.12	0	0.68
Insulin levels	304	319	-0.01mIU/l	0.97	-0.40, 0.38	0	0.47
SBP	471	465	-0.30 mm Hg	0.68	-1.76, 1.15	13	0.3
DBP	471	465	-1.05 mm Hg	0.06	-2.15, 0.04	35	0.08
Oxidized LDL	98	96	-0.29 U/l	0.93	-6.99, 6.41	54	0.07

**Legend**: A negative mean difference suggests an improvement in the intervention group over the control group. Several other subgroup analyses were carried out, but on measurements other than main lipid measurements. The analysis results can be found in Appendix A4.

## 4 Discussion

In this systematic review and meta-analysis, we evaluated the effects of interventions with berries high in anthocyanins for their ability to improve blood lipid profiles in MetS patients. HDL, LDL, and TC were evaluated as well as other parameters which are abnormal in MetS patients, including fasting glucose, Hb1Ac, insulin levels, blood pressure, and OX-LDL [77].

The meta-analysis showed a statistically significant decrease in LDL concentrations in the pooled data from all intervention groups compared with placebo (p=0.04). This finding correlates with the conclusions drawn by L. Yang et al. 2017, who reported a significant improvement in LDL [78]. However, the mean change in the present analysis was only 3.21 mg/dl and cannot be regarded as clinically relevant with regards to cardiovascular health [79]. Significant heterogeneity was present in the pooled intervention group, suggesting a significant variation between the studies. No other statistically significant differences were found for the 21 pooled studies (for HDL or TC).

Regarding fasting glucose, Hb1Ac, insulin levels, blood pressure, OX-LDL, and BMI end values, the only statistically significant difference seen was for BMI (-0.69 kg/m2, p=0.02, for the intervention group). A mean decrease of -0.69 kg/m2in BMI following an intervention may not be clinically relevant in terms of cardiovascular health. Also, as only 8 interventions were included, a more conclusive result may be obtained if more studies were available (I2 = 0%).

Fasting glucose and diastolic blood pressure remained unchanged, with p-values of 0.09 and 0.06 respectively. Statistically significant results were observed regarding HDL, LDL, and TC following an intervention with cranberries or for interventions lasting between four to six weeks; improvements of 2.01 mg/dl and 1.78 mg/dl were seen in the intervention groups, respectively (p=0.007 and p=0.03, respectively). An increase of one mg/dl in HDL has been shown to reduce the risk of CVD by 2% - 3% [80,81].

We found a statistically significant result regarding LDL levels when pooling all studies with freeze-dried strawberry interventions. In this case, LDL levels decreased in the intervention group by a mean of 11.04 mg/dl, p=0.002. It has been shown that decreasing LDL by 38.68 mg/dl reduces the risk of CHD by 23%. Hence, a reduction of 11.04 mg/dl, as observed in the present analysis, is likely to exert substantial improvements on CVD risk in MetS patients [82]. However, it is also essential to evaluate the ratio of LDL/HDL as elevated serum LDL/HDL ratio was independently associated with increased risk of CVD [83].

Finally, we found a statistically significant difference in TC levels after intervention with freeze-dried strawberries, with lower levels in the intervention group. The mean difference was -12.20 mg/dl (p<0.00001). In this regard, freeze-drying is a powerful method of increasing the concentration of nutrients and bioactive compounds by removing water from the berries. This may have had a significant effect on the results.

There are common limitations to meta-analyses when working with dietary interventions that must be considered when interpreting the results. Small sample sizes can cause I2 values to be low, even when studies are different. This particularly applies to subgroup analyses in the present study, where sample sizes were small indicating large heterogeneity. The large heterogeneity observed in some of these analyses maybe the consequence of different interventions, study designs, sample sizes, durations, and groups of participants.

Another major limitation to dietary interventions is the placebo product. It is of great importance to produce a placebo that is of similar appearance, texture, and flavor as the intervention product. A great variety of natural flavors and colorings are derived from fruits, especially fruits that are high in anthocyanins. Unless the studies have analyzed the placebo product with regards to anthocyanins, it is possible that the placebo could produce unwanted effects, although considering the low concentrations used for these purposes it must be regarded as negligible.

The time of the year also can influence findings; seasonal changes in dietary habits may have affected the outcome of the interventions [84]. Thus, it is not possible to attribute any of the tendencies or changes to anthocyanins alone. Anthocyanin-rich berries are also rich in vitamins and nutrients, carrying several bioactive components that potentially improve cardiovascular health in a synergistic way [85,86]. Vitamin C, vitamin E, and carotenoids are all found in berries and are known for their antioxidant capacities [87]. These bioactive compounds may act in a synergistic way with anthocyanins to achieve the positive results. Therefore, the observed results should be interpreted with caution. Moreover, there are also differences in the composition of bioactive substances between different varieties of berries, the conditions in which they are cultivated, and climate conditions, which may have introduced another bias into the meta-analysis. We cannot exclude the possibility that other bioactive compounds (e.g., fiber type and content) explain some of the beneficial effects detected by this meta-analysis.

Along similar lines, studies show that anthocyanin bioavailability is as low as 1%, suggesting that the observed beneficial effects cannot be attributed to anthocyanins, but to other bioactive components in the berries [88]. Likewise, some studies suggest that ingested anthocyanins are converted to bioavailable polyphenols by the gut microbiota, which may contribute to the positive effects found in the present meta-analysis [89-91].

Finally, vegetables and fruits other than those included in the studies also contain anthocyanins. Hence, the participants, including those in the placebo group, would have ingested anthocyanins from other sources. Therefore, it is difficult to say whether the anthocyanin in question was obtained from the interventions or from their usual daily diet. It would have been beneficial if all placebo groups had not been allowed to eat anthocyanin-containing food items to strengthen the results associated with anthocyanins in the diet.

Also, MetS is more common in elderly than in young adults and general nutrient bioavailability is poorer in the elderly than in young. Therefore, it would have been beneficial to have studies that discriminate on age so that it would be possible to determine how effective anthocyanins are relative to age [92].

The strength of this meta-analysis is the broad search for articles using MESH and EM-Tree terms. Moreover, none of the interventions in the metaanalysis had side effects or caused dangerously high levels of oxidative stress (measured by OX-LDL levels), blood lipids, or other parameters investigated.

A future perspective maybe to investigate anthocyanins in a more exclusive environment, i.e., only anthocyanin extracts should be investigated. This would enable a more robust result with regards to anthocyanins’ health benefits and exclude synergistic effects occurring with food matrices. Another approach would be to increase both the bioavailability and concentration of anthocyanins and other phenolic compounds in berries or berry products. This was shown by X. Du and A. D. Myracle in 2018 who changed the anthocyanin profile and raised the antioxidant capacity of aronia berries though kefir fermentation [93]. Therefore, it might be of interest to ferment berries or berry products and conduct an intervention with these products to evaluate their effects on the presently investigated parameters.

Another future perspective may be to use freeze- dried berries because promising in vivo results have been observed. A 2013 study by L. Brader et al. showed that following ingestion of 5 g/d freeze-dried bilberry powder for 8 weeks, a 60% reduction in LDL levels was seen in diabetic rats [94]. However, these results must be interpreted with caution as rodents in general have a different lipid metabolism than humans [95]. However, it still might be worth evaluating similar studies in humans with T2DM through a meta-analysis.

### 4.1 Conclusions

In this systematic review and meta-analysis, we investigated the effects of berries high in anthocyanins and their ability to improve the cardiovascular health of MetS patients. The overall analysis with all 25 interventions and more than 1300 participants showed no statistically significant rise in HDL levels. However, an increase was found in the cranberry intervention, and when the intervention period lasted 4-6 weeks. We found a statistically significant reduction in LDL in the intervention group. We found no statistically significant decrease in TC for pooled interventions.

The freeze-dried strawberry interventions also resulted in a significant decrease in TC and LDL. Hence, the data in this meta-analysis showed correlations between anthocyanin consumption and improvements in certain lipid biomarkers related to MetS. More data and studies are needed to develop recommendations regarding consumption of anthocyanin-rich berries for MetS, as effects from other bioactive compounds found in the berries may have interfered with the results in the present analysis.

## Abbreviations

CVD: cardiovascular disease

DBP: diastolic blood pressure

HDL: high-density lipoprotein

IL-6: interleukin6

IL-12: interleukin12

LDL: low-density lipoprotein

MetS: metabolic syndrome

OX-LDL: oxidized low-density lipoprotein

SBP: systolic blood pressure

T2DM: type 2 diabetes mellitus

TC: total cholesterol

TG: triglycerides

TNF-α: tumor necrosis factor a

## References

[1] Grundy SM, Hansen B, Smith SC, Cleeman JI, Kahn RA. Clinical Management of Metabolic Syndrome. Circulation 2004. 109(4):551-556. 14757684 10.1161/01.CIR.0000112379.88385.67

[2] Saklayen MG. The Global Epidemic of the Metabolic Syndrome. Curr Hypertens Rep 2018. 20(2):12. 29480368 10.1007/s11906-018-0812-z PMC5866840

[3] Popkin BM, Adair LS, Ng SW. Global nutrition transition and the pandemic of obesity in developing countries. Nutr Rev 2012. 70(1):3-21. 22221213 10.1111/j.1753-4887.2011.00456.x PMC3257829

[4] VanWormer JJ, Boucher JL, Sidebottom AC, Sillah A, Knickelbine T. Lifestyle changes and prevention of metabolic syndrome in the Heart of New Ulm Project. Prev Med rep 2017. 6:242-245. 28377851 10.1016/j.pmedr.2017.03.018 PMC5377429

[5] Babio N, Bullo M, Basora J, Martínez-González MA, Fernández-Ballart J, Márquez-Sandoval F, Molina C, Salas-Salvadó J. Adherence to the Mediterranean diet and risk of metabolic syndrome and its components. Nutr Metab Cardiovasc Dis 2009. 19(8):563-570. 19176282 10.1016/j.numecd.2008.10.007

[6] Kastorini CM, Milionis HJ, Esposito K, Giugliano D, Goudevenos JA, Panagiotakos DB. The effect of Mediterranean diet on metabolic syndrome and its components: a meta-analysis of 50 studies and 534,906 individuals. J Am Coll Cardiol 2011. 57(11):1299-1313. 21392646 10.1016/j.jacc.2010.09.073

[7] Tuso P, Stoll SR, Li WW. A plant-based diet, atherogenesis, and coronary artery disease prevention. Perm J 2015. 19(1):62-67. 25431999 10.7812/TPP/14-036 PMC4315380

[8] Qin Y, Xia M, Ma J, Hao Y, Liu J, Mou H, Cao L, Ling W. Anthocyanin supplementation improves serum LDL-and HDL-cholesterol concentrations associated with the inhibition of cholesteryl ester transfer protein in dyslipidemic subjects. Am J Clin Nutr 2009. 90(3):485-492. 19640950 10.3945/ajcn.2009.27814

[9] Zhu Y, Huang X, Zhang Y, Wang Y, Liu Y, Sun R, Xia M. Anthocyanin supplementation improves HDL-associated paraoxonase 1 activity and enhances cholesterol efflux capacity in subjects with hypercholesterolemia. J Clin Endocrinol Metab 2014. 99(2):561-569. 24285687 10.1210/jc.2013-2845

[10] Espinola-Klein C, Rupprecht HJ, Bickel C, Post F, Genth-Zotz S, Lackner K, Munzel T, Blankenberg S. Impact of metabolic syndrome on atherosclerotic burden and cardiovascular prognosis. Am J Cardiol 2007. 99(12):1623-1628. 17560864 10.1016/j.amjcard.2007.01.049

[11] Suh S, Baek J, Bae JC, Kim KN, Park MK, Kim DK, Cho NH, Lee MK. Sex factors in the metabolic syndrome as a predictor of cardiovascular disease. Endocrinol Metab 2014. 29(4):522-529. 10.3803/EnM.2014.29.4.522 PMC4285030 25559575

[12] Harchaoui K, Visser ME, Kastelein JJP, Stroes ES, Dallinga-Thie GM. Triglycerides and cardiovascular risk. Curr Cardiol Rev 2009. 5(3):216-222. 20676280 10.2174/157340309788970315 PMC2822144

[13] Abdullah SM, Defina LF, Leonard D, Barlow CE, Radford NB, Willis BL, Rohatgi A, McGuire DK, de Lemos JA, Grundy SM, et al. Long-Term Association of Low-Density Lipoprotein Cholesterol With Cardiovascular Mortality in Individuals at Low 10-Year Risk of Atherosclerotic Cardiovascular Disease. Circulation 2018. 138(21):2315-2325. 30571575 10.1161/CIRCULATIONAHA.118.034273

[14] Shah R, Murthy VL, Abbasi SA, Blankstein R, Kwong RY, Goldfine AB, Jerosch-Herold M, Lima JAC, Ding J, Allison MA. Visceral adiposity and the risk of metabolic syndrome across body mass index: the MESA Study. JACC Cardiovasc Imag 2014. 7(12):1221-1235. 10.1016/j.jcmg.2014.07.017 PMC4268163 25440591

[15] Monteiro R, Azevedo I. Chronic inflammation in obesity and the metabolic syndrome. Mediators Inflamm 2010. 289645. 20706689 10.1155/2010/289645 PMC2913796

[16] Christen T, Trompet S, Rensen PCN, Dijk KW Van, Lamb HJ, Jukema JW, Rosendaal FR, Cessie S, Mutsert R De. Nutrition, Metabolism & Cardiovascular Diseases The role of inflammation in the association between overall and visceral adiposity and subclinical atherosclerosis. Nutr Metab Cardiovasc Dis 2019. 29(7):728-735. 31138500 10.1016/j.numecd.2019.03.010

[17] Alvehus M, Burén J, Sjöström M, Goedecke J, Olsson T. The human visceral fat depot has a unique inflammatory profile. Obesity 2010. 18(5):879-883. 20186138 10.1038/oby.2010.22

[18] Cao YL, Hu CZ, Meng X, Wang DF, Zhang J. Expression of TNF-alpha protein in omental and subcutaneous adipose tissue in obesity. Diabetes Res Clin Pract 2008. 79(2):214-219. 17935818 10.1016/j.diabres.2007.08.030

[19] Fontana L, Eagon JC, Trujillo ME, Scherer PE, Klein S. Visceral Fat Adipokine Secretion Is Associated With Systemic Inflammation in Obese Humans. Diabetes 2007. 56(4): 10101013. 10.2337/db06-1656 17287468

[20] Mohammadi M, Gozashti MH, Aghadavood M, Mehdizadeh MR, Hayatbakhsh MM. Clinical Significance of Serum IL-6 and TNF-α Levels in Patients with Metabolic Syndrome. Reports Biochem Mol Biol 2017.6(1):74-79. PMC5643447 29090232

[21] Ferrannini E, Natali A, Bell P, Cavallo-Perin P, Lalic N, Mingrone G. Insulin resistance and hypersecretion in obesity. European Group for the Study of Insulin Resistance (EGIR). J Clin Invest 1997. 100(5):1166-1173. 9303923 10.1172/JCI119628 PMC508292

[22] Ferrannini E, Haffner SM, Mitchell BD, Stern MP. Hyperinsulinaemia: the key feature of a cardiovascular and metabolic syndrome. Diabetologia 1991. 34(6):416-422. 1884900 10.1007/BF00403180

[23] Olivecrona G, Olivecrona T. Triglyceride lipases and atherosclerosis. Curr Opin Lipidol 1995. 6(5):291-305. 8520852 10.1097/00041433-199510000-00009

[24] Beisiegel U. New aspects on the role of plasma lipases in lipoprotein catabolism and atherosclerosis. Atherosclerosis 1996. 124(1):1-8. 8800489 10.1016/0021-9150(95)05792-7

[25] Marseglia L, Manti S, D’Angelo G, Nicotera A, Párisi E, Di Rosa G, Gitto E, Arrigo T. Oxidative stress in obesity: a critical component in human diseases. Int J Mol Sci 2014. 16(1):378-400. 25548896 10.3390/ijms16010378 PMC4307252

[26] Rodríguez-Hernández H, Simental-Mendía LE, Rodríguez-Ramírez G, Reyes-Romero MA. Obesity and inflammation: epidemiology, risk factors, and markers of inflammation. Int J Endocrinol 2013. 678159. 23690772 10.1155/2013/678159 PMC3652163

[27] Leopold JA, Loscalzo J. Oxidative mechanisms and atherothrombotic cardiovascular disease. Drug Discov Today Ther Strateg 2008. 5(1):5-13. 21048889 10.1016/j.ddstr.2008.02.001 PMC2967043

[28] Xu S, Ogura S, Chen J, Little PJ, Moss J, Liu P. LOX-1 in atherosclerosis: biological functions and pharmacological modifiers. Cell Mol life Sci C 2013. 70(16):2859-2872. 10.1007/s00018-012-1194-z PMC4142049 23124189

[29] Ogura S, Kakino A, Sato Y, Fujita Y, Iwamoto S, Otsui K, Yoshimoto R, Sawamura T. Lox-1: the multifunctional receptor underlying cardiovascular dysfunction. Circ J Off J Japanese Circ Soc 2009. 73(11):1993-1999. 10.1253/circj.cj-09-0587 19801851

[30] Salvayre R, Auge N, Benoist H, Negre-Salvayre A. Oxidized low-density lipoprotein-induced apoptosis. Biochim Biophys Acta 2002. 1585(2-3):213-221. 12531556 10.1016/s1388-1981(02)00343-8

[31] Helkin A, Stein JJ, Lin S, Siddiqui S, Maier KG, Gahtan V. Dyslipidemia Part 1-Review of Lipid Metabolism and Vascular Cell Physiology. Vasc Endovascular Surg 2016. 50(2):107-118. 26983667 10.1177/1538574416628654

[32] Insull W. The Pathology of Atherosclerosis: Plaque Development and Plaque Responses to Medical Treatment. Am J Med 2009. 122(1):3-14. 10.1016/j.amjmed.2008.10.013 19110086

[33] Bartlett SM, Gibbons GF. Short-and longer-term regulation of very-low-density lipoprotein secretion by insulin, dexamethasone and lipogenic substrates in cultured hepatocytes. A biphasic effect of insulin. Biochem J 1988. 249(1):37-43. 3277620 10.1042/bj2490037 PMC1148662

[34] Malmström R, Packard CJ, Caslake M, Bedford D, Stewart P, Yki-Jarvinen H, Shepherd J, Taskinen MR. Defective regulation of triglyceride metabolism by insulin in the liver in NIDDM. Diabetologia 1997. 40(4):454-462. 9112023 10.1007/s001250050700

[35] Lewis GF, Uffelman KD, Szeto LW, Steiner G. Effects of acute hyperinsulinemia on VLDL triglyceride and VLDL apoB production in normal weight and obese individuals. Diabetes 1993. 42(6):833-842. 8495807 10.2337/diab.42.6.833

[36] Bae JH, Bassenge E, Kim KS, Kim YN, Kim KS, Lee HJ, Moon K, Lee MS, Park KY, Schwemmer M. Postprandial hypertriglyceridemia impairs endothelial function by enhanced oxidant stress. Atherosclerosis 2001. 155:517-523. 11254924 10.1016/s0021-9150(00)00601-8

[37] Hattori M, Nikolic-Paterson DJ, Miyazaki K, Isbel NM, Lan HY, Atkins RC, Kawaguchi H, Ito K Mechanisms of glomerular macrophage infiltration in lipid-induced renal injury. Kidney Int Suppl 1999. 71:47-50. 10.1046/j.1523-1755.1999.07112.x 10412736

[38] Cabrera de León A, Almeida González D, González Hernández A, Domínguez Coello S, Marrugat J, Juan Alemán Sánchez J, Brito Díaz B, Marcelino Rodríguez I, Pérez M del CR. Relationships between serum resistin and fat intake, serum lipid concentrations and adiposity in the general population. J Atheroscler Thromb 2014. 21(5):454-462. 24430788 10.5551/jat.22103

[39] Jiang C-Y, Wang W. Resistin aggravates the expression of proinflammatory cytokines in cerulein-stimulated AR42J pancreatic acinar cells. Mol Med Rep 2017. 15(1):502-506. 27959400 10.3892/mmr.2016.6027

[40] Farrell N, Norris G, Lee SG, Chun OK, Blesso CN. Function Anthocyanin-rich black elderberry extract improves markers of HDL function and reduces aortic cholesterol in hyperlipidemic mice. Food Funct 2015. 1(4):1278-1287. 10.1039/c4fo01036a 25758596

[41] Katherine TM, Joshua DB, Tanika NK, Jennifer ER, Patricia MK, Kristi R, Jing C, Jiang H. Global disparities of hypertension prevalence and control: a systematic analysis of population-based studies from 90 countries Circulation 2016. 134(6):441-450. 27502908 10.1161/CIRCULATIONAHA.115.018912 PMC4979614

[42] Soltani R, Hakimi M, Asgary S, Ghanadian SM, Keshvari M, Sarrafzadegan N. Evaluation of the Effects of Vaccinium arctostaphylos L. Fruit Extract on Serum Lipids and hs-CRP Levels and Oxidative Stress in Adult Patients with Hyperlipidemia: A Randomized, Double-Blind, Placebo-Controlled Clinical Trial. Nanji K, ed. Evidenc Based Complement Altern Med 2014. 217451. 10.1155/2014/217451 PMC3920853 24587807

[43] Lee IT, Chan YC, Lin CW, Lee WJ, Sheu WHH. Effect of cranberry extracts on lipid profiles in subjects with type 2 diabetes. Diabet Med 2008. 25(12):1473-1477. 19046248 10.1111/j.1464-5491.2008.02588.x

[44] Horbowicz M, Kosson R, Grzesiuk A, Dębski H. Anthocyanins of fruit and vegetables-their occurrence, analysis and role in human nutrition. Veg Crop Res Bull 2008. 68:5-22.

[45] Kim B, Bae M, Park YK, Ma H, Yuan T, Seeram NP, Lee JY. Blackcurrant anthocyanins stimulated cholesterol transport via post-transcriptional induction of LDL receptor in Caco-2 cells. Eur J Nutr 2018. 57(1):405-415. 28718016 10.1007/s00394-017-1506-z

[46] Li D, Zhang Y, Liu Y, Sun R, Xia M. Purified anthocyanin supplementation reduces dyslipidemia, enhances antioxidant capacity, and prevents insulin resistance in diabetic patients. J Nutr 2015. 145(4):742-748. 25833778 10.3945/jn.114.205674

[47] Guo H, Guo J, Jiang X, Li Z, Ling W. Cyanidin-3-O-P-glucoside, a typical anthocyanin, exhibits antilipolytic effects in 3T3-L1 adipocytes during hyperglycemia: Involvement of FoxOl-mediated transcription of adipose triglyceride lipase. FOOD Chem Toxicol 2012. 50(9):3040-3047. 22721980 10.1016/j.fct.2012.06.015

[48] Kao E, Tseng T, Lee H, Chan K, Wang C. Anthocyanin extracted from Hibiscus attenuate oxidized LDL-mediated foam cell formation involving regulation of CD36 gene. Chem Biol Interact 2009. 179:212-218. 19330881 10.1016/j.cbi.2009.01.009

[49] Zhang P, Chen F, Li D, Ling W, Guo H. A CONSORT-Compliant, Randomized, Double-Blind, Placebo-Controlled Pilot Trial of Purified Anthocyanin in Patients With Nonalcoholic Fatty Liver Disease. Medicine (Baltimore). 2015. 94(20):758. 10.1097/MD.0000000000000758 PMC4602870 25997043

[50] Jorgensen AB, Frikke-Schmidt R, Nordestgaard BG, Tybjsrg-Hansen A. Loss-of-function mutations in APOC3 and risk of ischemic vascular disease. N Engl J Med 2014. 371(1):32-41. 24941082 10.1056/NEJMoa1308027

[51] Walldius G, Jungner I. Apolipoprotein B and apolipoprotein A-I : risk indicators of coronary heart disease and targets for lipid-modifying therapy. J Intern Med 2004. 255(2):188-205. 14746556 10.1046/j.1365-2796.2003.01276.x

[52] Stull AJ, Cash KC, Johnson WD, Champagne CM, Cefalu WT. Bioactives in Blueberries Improve Insulin Sensitivity in Obese, Insulin-Resistant Men and Women. J Nutr 2010. 140(10):1764-1768. 20724487 10.3945/jn.110.125336 PMC3139238

[53] Takikawa M, Inoue S, Horio F, Tsuda T. Dietary anthocyanin-rich bilberry extract ameliorates hyperglycemia and insulin sensitivity via activation of AMP-activated protein kinase in diabetic mice. J Nutr 2010. 140(3):527-533. 20089785 10.3945/jn.109.118216

[54] Davinelli S, Bertoglio JC, Zarrelli A, Pina R, Scapagnini G. A Randomized Clinical Trial Evaluating the Efficacy of an Anthocyanin-Maqui Berry Extract (Delphinol®) on Oxidative Stress Biomarkers. J Am Coll Nutr 2015. 34(1):28-33. 26400431 10.1080/07315724.2015.1080108

[55] Khoo HE, Azlan A, Tang ST, Lim SM. Anthocyanidins and anthocyanins: colored pigments as food, pharmaceutical ingredients, and the potential health benefits. Food Nutr Res 2017. 61(1):1361779. 28970777 10.1080/16546628.2017.1361779 PMC5613902

[56] Federation ID. Metabolic Syndrome. 2006.

[57] Arnlov J, Sundstrom J, Ingelsson E, Lind L. Impact of BMI and the metabolic syndrome on the risk of diabetes in middle-aged men. Diabetes Care 2011. 34(1):61-65. 20852030 10.2337/dc10-0955 PMC3005442

[58] Curtis PJ, Van Der Velpen V, Berends L, Jennings A, Feelisch M, Umpleby AM, Evans M, Fernandez BO, Meiss MS, Minnion M, et al. Blueberries improve biomarkers of cardiometabolic function in participants with metabolic syndrome-results from a 6-month, double-blind, randomized controlled trial. Am J Clin Nutr 2019. 109(6):1535-1545. 31136659 10.1093/ajcn/nqy380 PMC6537945

[59] Aghababaee SK, Vafa M, Shidfar F, Tahavorgar A, Gohari M, Katebi D, Mohammadi V. Effects of blackberry (Morus nigra L.) consumption on serum concentration of lipoproteins, apo A-I, apo B, and high-sensitivity-C-reactive protein and blood pressure in dyslipidemic patients. J Res Med Sci 2015. 20(7):685-691. 10.4103/1735-1995.166227 PMC4638072 26622259

[60] Burton-freeman B, Linares A, Hyson D, Kappagoda T. Strawberry modulates LDL oxidation and postprandial lipemia in response to high-fat meal in overweight hyperlipidemic men and women. JAm Coll Nutr 2010. 29(1):46-54. 20595645 10.1080/07315724.2010.10719816

[61] Basu A, Betts NM, Nguyen A, Newman ED, Fu D, Lyons TJ. Freeze-Dried Strawberries Lower Serum Cholesterol and Lipid Peroxidation in Adults with Abdominal Adiposity and Elevated Serum Lipids. J Nutr 2014. 144(6):830-837. 24670970 10.3945/jn.113.188169 PMC4018947

[62] Novotny JA, Baer DJ, Khoo C, Gebauer SK, Charron CS. Cranberry Juice Consumption Lowers Markers of Cardiometabolic Risk, Including Blood Pressure and Circulating C-reactive Protein, Triglyceride, and Glucose Concentrations in Adults. J Nutr 2015. 145(6):1185-1193. 25904733 10.3945/jn.114.203190

[63] Paquette M, Larque ASM, Weisnagel SJ, Desjardins Y, Marois J, Pilon G, Dudonne S, Marette A, Jacques H. Strawberry and Cranberry Polyphenols Improve Insulin Sensitivity in Insulin-Resistant, Non-Diabetic Adults: A Parallel, Double-Blind, Controlled and Randomised Clinical Trial. Br J Nutr 2017. 117(4):519-531. 28290272 10.1017/S0007114517000393 PMC5426341

[64] Ruel G, Pomerleau S, Couture P, Lemieux S, Lamarche B, Couillard C. Low-calorie cranberry juice supplementation reduces plasma oxidized LDL and cell adhesion molecule concentrations in men. Br J Nutr 2008. 99(2):352-359. 17761017 10.1017/S0007114507811986

[65] Xie L, Vance T, Kim B, Lee SG, Caceres C, Wang Y, Hubert PA, Lee JY, Chun OK, Bolling BW. Aronia berry polyphenol consumption reduces plasma total and low-density lipoprotein cholesterol in former smokers without lowering biomarkers of inflammation and oxidative stress: a randomized controlled trial. Nutr Res 2017. 37:67-77. 10.1016/j.nutres.2016.12.007 28215316

[66] Zunino SJ, Parelman MA, Freytag TL, Stephensen CB, Kelley DS, Mackey BE, Woodhouse LR, Bonnel EL. Effects of dietary strawberry powder on blood lipids and inflammatory markers in obese human subjects. Br J Nutr 2012. 108(5):900-909. 22068016 10.1017/S0007114511006027

[67] Arevstrom L, Bergh C, Landberg R, Wu H, Rodriguez-mateos A, Waldenborg M, Magnuson A, Blanc S, Fröbert O. Freeze-dried Bilberries (Vaccinium myrtillus ) dietary supplement improves walking distance and lipids after myocardial infarction: an open-label randomized clinical trial. Nutr Res 2018. 62:13-22. 30803503 10.1016/j.nutres.2018.11.008

[68] Mulero J, Bernabe J, Cerda B, Garcia-viguera C, Moreno DA, Dolores M, Aviles F, Parra S, Abellan J, Zafrilla P. Variations on cardiovascular risk factors in metabolic syndrome after consume of a citrus-based juice. Clin Nutr 2012. 31(3):372-377. 22197455 10.1016/j.clnu.2011.11.014

[69] Zhu Y, Xia M, Yang Y, Liu F, Li Z, Hao Y, Mi M, Jin T, Ling W. Purified Anthocyanin Supplementation Improves Endothelial Function via NO-cGMP Activation in Hypercholesterolemic Individuals. Clin Chem 2011. 57(11):1524-1533. 21926181 10.1373/clinchem.2011.167361

[70] Basu A, Fu DX, Wilkinson M, Simmons B, Wu M, Betts NM, Du M, Lyons TJ. Strawberries decrease atherosclerotic markers in subjects with metabolic syndrome. Nutr Res 2010. 30(7):462-469. 20797478 10.1016/j.nutres.2010.06.016 PMC2929388

[71] Basu A, Betts NM, Ortiz J, Simmons B, Wu M, Lyons TJ. Low-energy cranberry juice decreases lipid oxidation and increases plasma antioxidant capacity in women with metabolic syndrome. Nutr Res 2011. 31(3):190-196. 21481712 10.1016/j.nutres.2011.02.003 PMC3075541

[72] Amani R, Moazen S, Shahbazian H, Ahmadi K, Jalali MT. Flavonoid-rich beverage effects on lipid profile and blood pressure in diabetic patients. World J Diabetes 2014. 5(6):962-968. 25512803 10.4239/wjd.v5.i6.962 PMC4265887

[73] Kanellos PT, Kaliora AC, Tentolouris NK, Argiana V, Perrea D, Kalogeropoulos N, Kountouri AM, Karathanos VT. A pilot, randomized controlled trial to examine the health outcomes of raisin consumption in patients with diabetes. Nutr 2014. 30(3):358-364. 10.1016/j.nut.2013.07.020 24262513

[74] Mirfeizi M, Tourzani ZM, Mirfeizi SZ, Jafarabadi MA, Rezvani HR, Afzali M. Controlling Type 2 Diabetes Mellitus With Herbal Medicines: A Triple-Blind Randomized Clinical Trial of Efficacy and Safety. J Diabetes 2016. 8(5):647-656. 26362826 10.1111/1753-0407.12342

[75] Javid AZ, Maghsoumi-norouzabad L, Ashrafzadeh E, Yousefimanesh HA, Zakerkish M, Angali KA. Impact of Cranberry Juice Enriched With Omega-3 Fatty Acids Adjunct With Nonsurgical Periodontal Treatment on Metabolic Control and Periodontal Status in Type 2 Patients With Diabetes With Periodontal Disease. J Am Coll Nutr 2018. 37(1):71-79. 29272211 10.1080/07315724.2017.1357509

[76] Eker ME, Aaby K, Budic-Leto I, Brncic SR, El SN, Karakaya S, Simsek S, Manach C, Wiczkowski W, Pascual-Teresa S de. A Review of Factors Affecting Anthocyanin Bioavailability: Possible Implications for the Inter-Individual Variability. Foods 2019. 9(1):2. 10.3390/foods9010002 PMC7023094 31861362

[77] Holvoet P, De Keyzer D, Jacobs Jr DR. Oxidized LDL and the metabolic syndrome. Future Lipidol 2008. 3(6):637-649. 19802339 10.2217/17460875.3.6.637 PMC2631666

[78] Yang L, Ling W, Du Z, Chen Y, Li D, Deng S, Liu Z, Yang L. Effects of Anthocyanins on Cardiometabolic Health: A Systematic Review and Meta-Analysis of Randomized Controlled Trials. Adv Nutr 2017. 8(5):684-693. 28916569 10.3945/an.116.014852 PMC5593100

[79] Khera AV, Plutzky J. Management of low levels of high-density lipoprotein-cholesterol. Circulation 2013. 128(1):72-78. 23817482 10.1161/CIRCULATIONAHA.112.000443 PMC4231714

[80] Gordon DJ, Probstfield JL, Garrison RJ, Neaton JD, Castelli WP, Knoke JD, Jacobs DRJ, Bangdiwala S, Tyroler HA. High-density lipoprotein cholesterol and cardiovascular disease. Four prospective American studies. Circulation 1989. 79(1):8-15. 2642759 10.1161/01.cir.79.1.8

[81] Di Angelantonio E, Sarwar N, Perry P, Kaptoge S, Ray KK, Thompson A, Wood AM, Lewington S, Sattar N, Packard CJ, et al. Major lipids, apolipoproteins, and risk of vascular disease. >JAMA 2009. 302(18):1993-2000. 19903920 10.1001/jama.2009.1619 PMC3284229

[82] Scirica BM, Cannon CP. Treatment of Elevated Cholesterol. Circulation 2005. 111(21):360-363. 10.1161/CIRCULATIONAHA.105.539106 15927980

[83] Kunutsor SK, Zaccardi F, Karppi J, Kurl S, Laukkanen JA. Is High Serum LDL/HDL Cholesterol Ratio an Emerging Risk Factor for Sudden Cardiac Death? Findings from the KIHD Study. JAtheroscler Thromb 2017. 24(6):600-608. 27784848 10.5551/jat.37184 PMC5453685

[84] Shahar DR, Yerushalmi N, Lubin F, Froom P, Shahar A, Kristal-Boneh E. Seasonal variations in dietary intake affect the consistency of dietary assessment. Eur J Epidemiol 2001. 17(2):129-133. 11599685 10.1023/a:1017542928978

[85] Shixian Q, Dai Y, Kakuda Y, Shi J, Mittal G, Yeung D, Jiang Y. Synergistic Anti-Oxidative Effects of Lycopene with Other Bioactive Compounds. Food Rev Int 2005. 21(3):295-311.

[86] Zhao CN, Meng X, Li Y, Li S, Liu Q, Tang GY, Li HB. Fruits for Prevention and Treatment of Cardiovascular Diseases. Nutr 2017. 9(6):598. 10.3390/nu9060598 PMC5490577 28608832

[87] Debreceni B, Debreceni L. Role of vitamins in cardiovascular health and disease. Res Reports Clin Cardiol 2014. 283.

[88] Manach C, Williamson G, Morand C, Scalbert A. Bioavailability and bioefficacy of polyphenols in humans. I. Review of 97 bioavailability studies. Am J Clin Nutr 2005. 81:230-242. 10.1093/ajcn/81.1.230S 15640486

[89] Oodward GW, Roon PK, Cassidy AC, Colin K. Anthocyanin Stability and Recovery: Implications for the Analysis of Clinical and Experimental Samples. J Agric Food Chem 2009. 57(12):5271-5278. 19435353 10.1021/jf900602b

[90] Forman HJ, Davies KJA, Ursini F. Free Radical Biology and Medicine How do nutritional antioxidants really work : Nucleophilic tone and para-hormesis versus free radical scavenging in vivo. Free Radic Biol Med 2014. 66:24-35. 23747930 10.1016/j.freeradbiomed.2013.05.045 PMC3852196

[91] Fraga CG, Oteiza PI, Galleano M. Plant bioactives and redox signaling: (-)-Epicatechin as a paradigm. Mol Aspects Med 2018. 61:31-40. 29421170 10.1016/j.mam.2018.01.007

[92] Pray L, Boon C, Miller EA, Laura P. Providing Healthy and Safe Foods As We Age: Workshop Summary. Natl Acad Press 2010. 21391340

[93] Du X, Myracle AD. Fermentation alters the bioaccessible phenolic compounds and increases the alpha-glucosidase inhibitory effects of aronia juice in a dairy matrix following in vitro digestion. Food Funct 2018. 9(5):2998-3007. 29774337 10.1039/c8fo00250a

[94] Brader L, Overgaard A, Christensen LP, Jeppesen PB, Hermansen K. Polyphenol-rich bilberry ameliorates total cholesterol and LDL-cholesterol when implemented in the diet of Zucker diabetic fatty rats. Rev Diabet Stud 2013. 10(4):270-282. 24841880 10.1900/RDS.2013.10.270 PMC4160013

[95] Bergen WG, Mersmann HJ. Comparative Aspects of Lipid Metabolism: Impact on Contemporary Research and Use of Animal Models. J Nutr 2005. 135(11):2499-2502. 16251600 10.1093/jn/135.11.2499

